# The role of the RNA chaperone Hfq in *Haemophilus influenzae* pathogenesis

**DOI:** 10.1186/1471-2180-13-134

**Published:** 2013-06-16

**Authors:** Randy J Hempel, Daniel J Morton, Thomas W Seale, Paul W Whitby, Terrence L Stull

**Affiliations:** 1 Department of Pediatrics, University of Oklahoma Health Sciences Center, Oklahoma City, OK 73104, USA; 2 Department of Microbiology and Immunology, University of Oklahoma Health Sciences Center, Oklahoma City, OK 73104, USA

**Keywords:** Haemophilus, Hfq, Pathogenesis, Otitis media, Bacteremia

## Abstract

**Background:**

The RNA binding protein Hfq of *Haemophilus influenzae* is highly homologous to Hfq from other bacterial species. In many of these other bacteria, Hfq affects the expression of a broad range of genes and enhances the ability to respond to stressful environments. However, the role of Hfq in *H*. *influenzae* is unknown.

**Results:**

Deletion mutants of *hfq* were generated in the nontypeable *H*. *influenzae* strains R2866 and 86-028NP to assess the role of Hfq in these well characterized but genotypically and phenotypically divergent clinical isolates. A deletion mutation of *hfq* had no effect on growth of *H*. *influenzae* in nutrient rich media and had no effect on survival in several stressful conditions *in vitro*. However, the mutation resulted in a reduced ability to utilize heme from hemoglobin. The mutant and wild type strains were assessed for virulence and competitive fitness in models of invasive disease and otitis media. In the chinchilla model of otitis media, the *hfq* mutant of 86-028NP exhibited impaired competitive fitness when compared to its wild type progenitor but exhibited no apparent defect in virulence. In the infant rat model, deletion of *hfq* in R2866 resulted in reduced bacterial titers in blood and a shorter duration of infection when compared to the wild type strain in the competitive fitness study.

**Conclusion:**

We conclude that Hfq is involved in the utilization of essential nutrients and facilitates infection by *H*. *influenzae*.

## Background

*H*. *influenzae* is a fastidious, Gram-negative, opportunistic pathogen that belongs to the family Pasteurellaceae and is a common commensal in the nasopharynx of humans [1,2]. *H*. *influenzae* is a causative agent of both invasive and non-invasive diseases including bacteremia, meningitis, respiratory infections, and otitis media [1]. Invasive disease may be caused by either encapsulated or nonencapsulated strains [3], whereas non-invasive diseases are primarily caused by nonencapsulated, nontypeable *H*. *influenzae*[4].

Like most other bacteria, *H*. *influenzae* requires iron for growth but it also has an absolute requirement for a porphyrin source, in the form of protoporphyrin IX (PPIX) or heme, to grow aerobically [5]. This requirement for a porphyrin source is due to the lack of enzymes required to synthesize the protoporphyrin ring. Therefore, *H*. *influenzae* must acquire heme from host sources in order to establish and sustain an infection [6]. Potential sources of heme in the human host are limited; heme is generally intracellular, bound by hemoglobin or other heme-containing proteins, and there is no significant source of PPIX [7,8]. *H*. *influenzae* has evolved multiple mechanisms to counter and exploit host mechanisms for sequestering heme from invading pathogens [9]. Although many of these mechanisms are transcriptionally upregulated in response to iron and heme restriction, the specific regulation of many of these systems is largely uncharacterized in *H*. *influenzae*[10,11].

The RNA-binding protein Hfq is an important regulator of gene transcription, including the transcription of iron responsive genes, in many bacterial pathogens such as *Escherichia coli*, *Neisseria meningitidis*, and *Salmonella enterica*[12-14]. The Hfq protein was originally described as a host factor required for the synthesis of bacteriophage Qβ RNA in *E*. *coli* and belongs to the Sm and Sm-like family of proteins that are found in both prokaryotes and eukaryotes [15,16]. Hfq has been described as an integral component of post-transcriptional gene regulation because of its ability to bind both small regulatory RNAs (sRNAs) and their mRNA target, altering the stability of the bound mRNA [17]. Several studies have revealed the role of Hfq and sRNAs in post-transcriptional regulation of iron responsive genes [18-20]. Hfq is found in many bacterial pathogens and is a pleiotropic gene regulator; mutants exhibit phenotypes including defects in virulence, growth rates, stress tolerance and biofilm formation [21]. The phenotypes of *hfq* mutants vary greatly between bacterial species because of the wide array of RNA with which Hfq interacts [17].

Here we report the characterization of a deletion mutant of *hfq* in *H*. *influenzae*. We demonstrate *in vitro* that Hfq is important in modulating the utilization of heme from hemoglobin. Further we show that Hfq plays a role in pathogenesis in the infant rat and chinchilla models of disease. Thus, Hfq may be modulating nutrient utilization systems that allow *H*. *influenzae* to better adapt to niches within the host during infection.

## Methods

### Bacterial strains and growth conditions

Nontypeable *H*. *influenzae* strain R2866 is a clinical isolate from the blood of an immunocompetent pediatric patient with clinical signs of meningitis following acute OM [22]. Nontypeable *H*. *influenzae* strain 86-028NP was isolated from the nasopharynx of a child being treated for chronic OM who underwent tympanostomy and tube insertion [23,24]. *H*. *influenzae* strains were routinely grown on chocolate agar with bacitracin at 37°C. *H*. *influenzae* was also cultured on brain heart infusion (BHI) agar or in BHI broth supplemented with 10 μg mL^-1^ heme and 10 μg mL^-1^ β-NAD (supplemented BHI; sBHI) or BHI supplemented with 10 μg mL^-1^ β-NAD (heme deplete BHI; hdBHI). The antibiotics spectinomycin (200 μg mL^-1^) and chloramphenicol (2.0 μg mL^-1^) were used when appropriate.

### Heme sources

Human hemoglobin and heme (as hemin) were purchased from Sigma. Stock heme solutions were prepared at 1.0 mg mL^-1^ heme in 4% v/v triethanolamine as previously described [25]. Hemoglobin was dissolved in water immediately before use.

### Construction of the *hfq* mutant

A deletion mutant lacking the entire *hfq* gene was constructed using two pairs of primers to amplify regions upstream and downstream of *hfq* by PCR using strain R2866 DNA as template. Primer pair Hfq_US1 (GAATTCGATTTGTTAGGAAAGCCTGCC) and Hfq_US2 (GGATCCGCGGTTGAAAATTCTCAGGAAA) was used to amplify an 867-bp fragment upstream of *hfq* with EcoRI and BamHI restriction sites engineered into the primers, respectively, to allow for directional subcloning. Hfq_DS1 (GGATCCAGAAACGAGTTGTCTCCGTG) and Hfq_DS2 (AAGCTTCGAAGTGCGAGTAAACAAAGGC) were used to amplify an 869-bp fragment downstream of *hfq* with BamHI and HindIII restriction sites incorporated into the primers, respectively. The PCR products were cloned into the TA cloning vector pCR2.1-TOPO (Invitrogen) and the cloned sequences were confirmed by DNA sequencing. After the DNA sequences were confirmed, the upstream and downstream fragments were directionally subcloned into pUC19N to give a plasmid containing the fragments abutting each other with a unique BamHI site between the two products. The BamHI site was used to insert a 1214-bp fragment containing a spectinomycin resistance cassette from pSPECR [26], and produced the mutagenic construct pRH30.

The plasmid construct pRH30 was used to transform *H*. *influenzae* strains R2866 and 86-028NP by the static-aerobic method as previously described [27] and transformants were selected on spectinomycin. Transformants resistant to spectinomycin were confirmed using PCR.

### Complementation of the *hfq* deletion mutant

For complementation of the *hfq* deletion a region encompassing 450-bp upstream to 286-bp downstream of *hfq* was amplified from strain R2866 using primers Hfqcmp_fwd (GGATCCACAAAGTGCGGTGATTTCTTTGGAT) and Hfqcmp_rev (TCTAGAGAATTATCTAGCGGAGAGCGCATTG). The primers Hfqcmp_fwd and Hfqcmp_rev had respectively BamHI and XbaI restriction sites incorporated to allow for subcloning. The PCR product was cloned into pCR2.1-TOPO and subsequently subcloned into the vector pASK5 to yield pRH38. The vector pASK5 was designed to allow complementation of gene disruptions in *H*. *influenzae* by insertion of a gene in the nonessential outer-membrane protein OmpP1 locus and has been successfully used in our laboratory [28-33]. The plasmid pRH38 was used to transform the R2866 ∆∆*hfq* strain, HI2206, to chloramphenicol resistance to yield strain HI2210. Correct insertion of the complementation construct was confirmed by PCR.

### Primer extension analysis

Primer extension analysis was performed as previously described [34,35]. RNA was purified from a *H*. *influenzae* culture grown to mid-log phase in sBHI using the Qiagen RNeasy Mini Kit. The RNA was DNase treated and the integrity was verified by agarose gel electrophoresis. A total of 10 μg of RNA was used to synthesize cDNA using a 6-carboxyfluorescein (FAM)-labeled primer, hfq-PE (ATTGATACAGGAATGCGTTCACGAC). The hfq-PE primer was added to the RNA and they were incubated at 70°C for 10 min and chilled on ice before being incubated at 65°C for 2 min. The mixture was incubated at 42°C and the cDNA synthesis reagents [4 μl 10× reverse transcriptase (RT) buffer, 8 μL 25 mM MgCl_2_, 4 μL 10 mM deoxynucleoside triphosphates (dNTPs), 1 μl RNase inhibitor, 2 μL Multiscribe RT (Applied Biosystems)] were added to the mixture, incubated for 2 h, and ethanol precipitated. The sizing of the cDNA fragments was performed by the Laboratory for Genomics and Bioinformatics at the University of Oklahoma Health Sciences Center. Analysis of the fragments was done using Peak Scanner software (Applied Biosciences).

### Growth studies with *H*. *Influenzae*

Growth studies were performed using the Bioscreen C Microbiology Reader (Oy Growth Curves AB Ltd., Helsinki, Finland) as previously described [36,37]. *H*. *influenzae* strains were grown for 16 hours on chocolate agar with bacitracin and used to seed hdBHI broth cultures which were incubated for 3 h with shaking at 37°C. The 3 h cultures were pelleted by centrifugation, washed in phosphate buffered saline (PBS) containing 0.1% w/v gelatin, and resuspended to an optical density of 0.5 at 605 nm in the same buffer. The bacterial suspension was diluted by adding 1.0 mL into 5.0 mL of PBS containing 0.1% gelatin and was used to inoculate media for growth curves (approximate initial concentration of 200,000 cfu ml^-1^).

*In vitro* competition studies were performed by mixing equal numbers of the wild type and mutant strains (starting total of approximately 2 × 10^5^ cfu ml^-1^) in 50 ml of either sBHI or hdBHI supplemented with limiting concentrations of hemoglobin (5 μg ml^-1^. Bacterial counts were determined for the duration of the 28 hour experiment by plating samples using the track dilution method, as previously described [38], on sBHI or sBHI containing spectinomycin to allow enumeration of both strains.

### Chinchilla model of otitis media

Adult chinchillas (*Chinchilla lanigera*) with no signs of middle ear infection by either otoscopy or tympanometry at the beginning of the study were used. Animals were allowed to acclimate to the vivarium for at least 14 days prior to transbullar challenge. Animal procedures have been previously described in detail [39-41].

Two separate experiments, one to assess virulence and a second to assess competitive fitness, were performed in the chinchillas.

In the first experiment to compare virulence, two groups of 5 animals were challenged in both ears by transbullar injection with approximately 2,000 cfu of either strain 86-028NP or its *hfq* deletion mutant HI2207. Transbullar inocula were delivered in 300 μl 0.1% gelatin in PBS by direct injection into the superior bullae. Actual bacterial doses were confirmed by plate count. On days 4, 7, 11, and 14 post-challenge middle ear effusions (MEE) were collected by epitympanic tap as previously described [29]. Bacterial titers in recovered MEE were determined using the track dilution method.

In the second experiment, to assess competitive fitness, five animals were challenged in both ears transbullarly with a mixture containing equal numbers of 86-028NP and its *hfq* deletion mutant HI2207 (total of approximately 2,000 cfu). Epitympanic taps were performed on all ears on days 4, 7, 11, and 14 after nontypeable *H*. *influenzae* challenge. Recovered MEE were plated on sBHI and sBHI containing spectinomycin in order to determine the total bacterial titer and the titer of the mutant strain respectively.

### Rat model of bacteremia

The infant rat model for hematogeneous meningitis following intraperitoneal infection with *H*. *influenzae*[42] was used to compare the abilities of strains R2866 and the ∆*hfq* mutant, HI2206, to cause bacteremia.

Again two experiments were performed, one to assess virulence and a second to assess competitive fitness.

Specified pathogen free (SPF), timed-pregnant Sprague–Dawley rats (Harlan Sprague–Dawley) were received approximately five days prior to giving birth. These pregnant females were single housed on hardwood litter with *ad libitum* access to water and a standard pelleted food (Purina Lab Rodent Diet 5001). They were maintained on a 12 hour light–dark cycle in separate forced air cubicles in a bio-containment facility to prevent cross-contamination. Newborn pups from different mothers were pooled and randomly reassigned to the mothers (n=10 pups per female).

In the first experiment to assess virulence two groups of ten 5-day-old infant rats were infected with 100,000 cfu of either R2866 or the corresponding *hfq* mutant HI2206 suspended in 100 μl PBS by intraperitoneal injection. Inocula were prepared as previously described [43]. The dosage used to infect the rats was confirmed by plate count. Rats were examined for signs of infection (neurological symptoms: tremor, loss of righting ability, coma, rigidity; systemic symptoms: lethargy, anorexia, hypothermia) at 24-hour intervals. After placing the animals under anesthesia (gaseous isoflurane; Butler Animal Health Supply, Dublin, OH), cardiac puncture was used to obtain blood specimens on days 1, 2, 3, and 4 post-infection [42].

In the second experiment to assess competitive fitness a group of ten 5-day old rats was infected by intraperitoneal injection with a 1:1 mixed culture (WT:∆*hfq* or Complement:∆*hfq*) of 100,000 cfu of each strain in 100 μL PBS. Rats were examined for clinical signs of infection and bacteremia as described above in the virulence experiment.

The track dilution method was used to quantify bacteremia by serially diluting (0 to 10^-5^) whole-blood specimens freshly drawn in heparinized syringes with PBS. Aliquots of 10 μL from each dilution were plated in triplicate on sBHI agar, with or without the appropriate antibiotic in the case of the fitness study, and incubated at least 18 hours at 37°C for quantification.

### Ethics statement

All animal studies described herein were performed in strict accordance with the recommendations in the Guide for the Care and Use of Laboratory Animals (National Institutes of Health). Animal protocols were reviewed and approved by the Institutional Animal Care and Use Committee of the University of Oklahoma Health Sciences Center.

### Statistics

A Mann–Whitney test was performed on all *in vitro* growth data over the duration of the experiments using GraphPad Prism software version 5.0a (GraphPad Software, San Diego California USA, http://www.graphpad.com). Bacteremic titers from the *in vivo* studies were analyzed using a two-tailed Student t-test. A Fisher’s exact test and a one-sample t-test were performed to compare the competitive index. A *P* value <0.05 was taken as significant.

## Results and discussion

### Promoter and sequence analysis of *hfq* in *H*. *influenzae*

**Figure 1:**
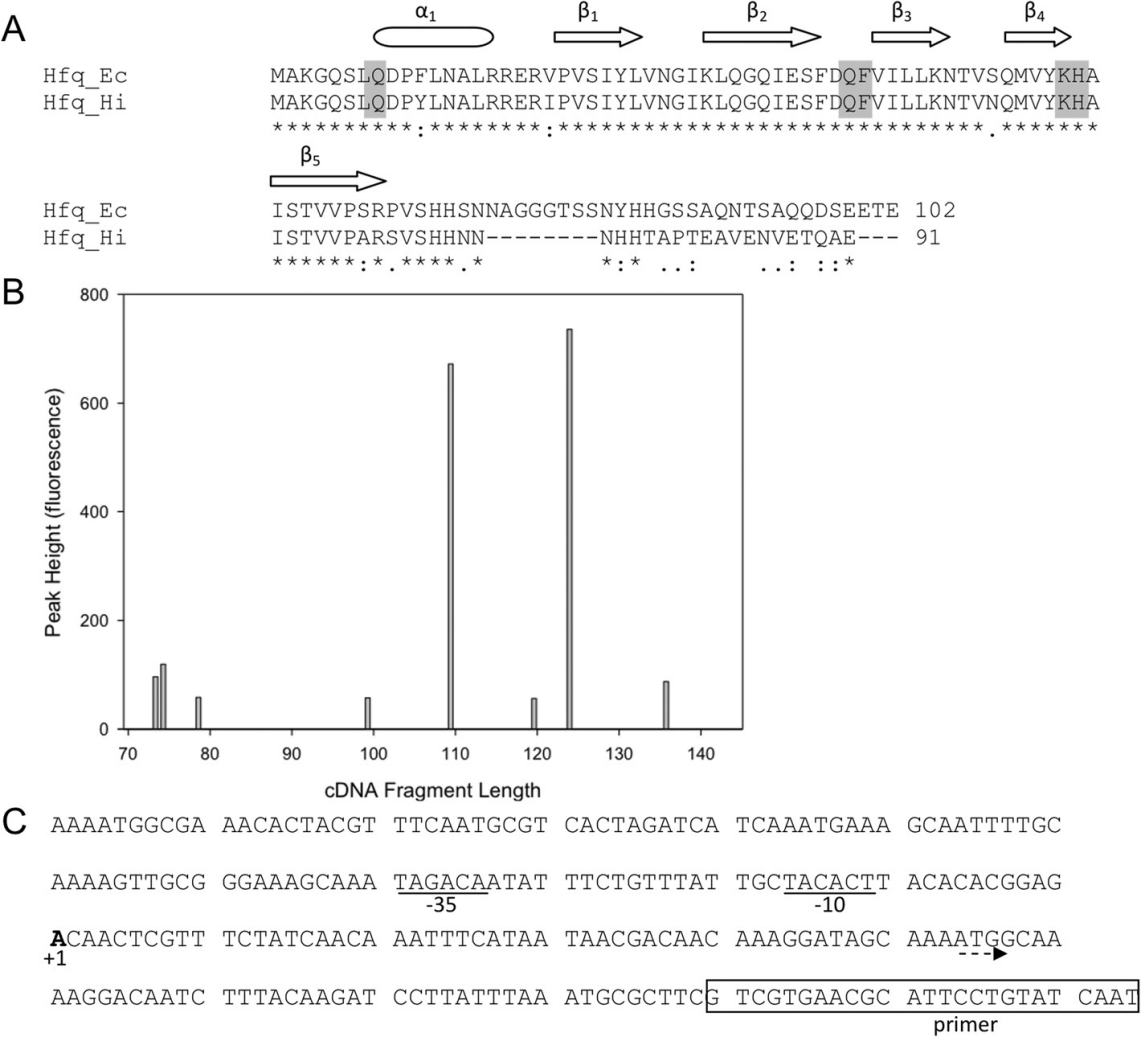
**Characterization of the *hfq *gene in *H. influenzae*.** (**A**) CLUSTALW alignment between the Hfq of *E*. *coli* (Hfq_Ec) and *H*. *influenzae*. Amino acids denoted by asterisks (*) are identical, colons (:) strongly similar and dots (.) weakly similar. The secondary structure of the *E*. *coli* Hfq is indicated above the sequence and dashed-line boxes denote the Sm1 and Sm2 motifs. The shaded boxes are residues that are important in RNA binding by the Hfq of *S*. *aureus* and the two signature motifs of Hfq are underlined. This was modified from the figure of Nielson et al. [44]. (**B**) Fluorescent intensities of primer extension products synthesized from *H*. *influenzae* RNA. (**C**) Sequence of the transcription start site (+1) and the proposed promoter region for the *H*. *influenzae* Hfq gene. The sequence complementary to the primer used for primer extension is boxed, the transcription start site is boldfaced, and the putative −10 and −35 promoter sequences are underlined.

Hfq is encoded by the gene HI0411 in the *H*. *influenzae* reference strain Rd KW20 [GenBank: NC_000907] and consists of 91 amino acids and is at least 97% identical to Hfq in 20 sequenced strains of *H*. *influenzae*. Furthermore, it is 78% similar to the Hfq protein from *E*. *coli* and all residues that contribute to RNA binding in the latter species are conserved (Figure 1A) [44]. By comparison with HI0411, the Hfq protein in *E*. *coli* contains a longer C terminal extension. This C terminal extension is highly variable among different species of bacteria and does not contribute to the overall activity of Hfq [45]. The *hfq* gene is located on the lagging strand of the Rd KW20 genome and is downstream of the *tyrR* gene (encoding a transcriptional regulatory protein) and upstream of HI0412 (encoding 23S rRNA pseudouridylate synthase C). These same two genes also flank *hfq* in the *H*. *influenzae* strains used in the remainder of this study. The *hfq* gene is highly conserved among all sequenced strains of *H*. *influenzae*, an indication that this gene serves an important function in this species. This would suggest that *H*. *influenzae* also uses Hfq along with sRNAs to modulate gene expression, the posited role for Hfq in other prokaryotes.

Nontypeable *H*. *influenzae* strains R2866 and 86-028NP were selected for the studies described herein since both strains have each been well characterized both genetically and phenotypically. Both strains have also been extensively used in the animal models described herein [22,29,41,46-48]. Rd KW20 was not used for further study because it is considered an avirulent ‘laboratory strain’ of *H*. *influenzae* since it has lost the genes that encode the type d capsule and lacks adhesins that are necessary for nontypeable *H*. *influenzae* disease [49,50].

In several organisms the *hfq* gene is co-transcribed with the upstream gene *miaA* when that gene is present [51,52]. However, in bacterial species in which a gene other than *miaA* is upstream, *hfq* is not co-transcribed [53]. RT-PCR experiments performed in R2866 and 86-028NP indicated that *hfq* is not co-transcribed with either of the flanking genes (data not shown). Primer extension experiments were performed to identify the transcription start site and to help determine the location of the promoter. The major primer restriction product was 123 nt in length (Figure 1B), corresponding to an adenine transcriptional start site 53 nt upstream of the ATG start codon (Figure 1C).

Since the sequence of *hfq* is well conserved in experimentally relevant strains, *hfq* deletion mutants were constructed in order to study the role of Hfq in *H*. *influenzae*. Deletion mutants of the *hfq* genes of *H*. *influenzae* nontypeable strains R2866 and 86-028NP were successfully constructed and confirmed by PCR (data not shown) and were designated HI2206 and HI2207 respectively.

### *In vitro* growth characteristics of *H*. *influenzae hfq* mutants

**Figure 2:**
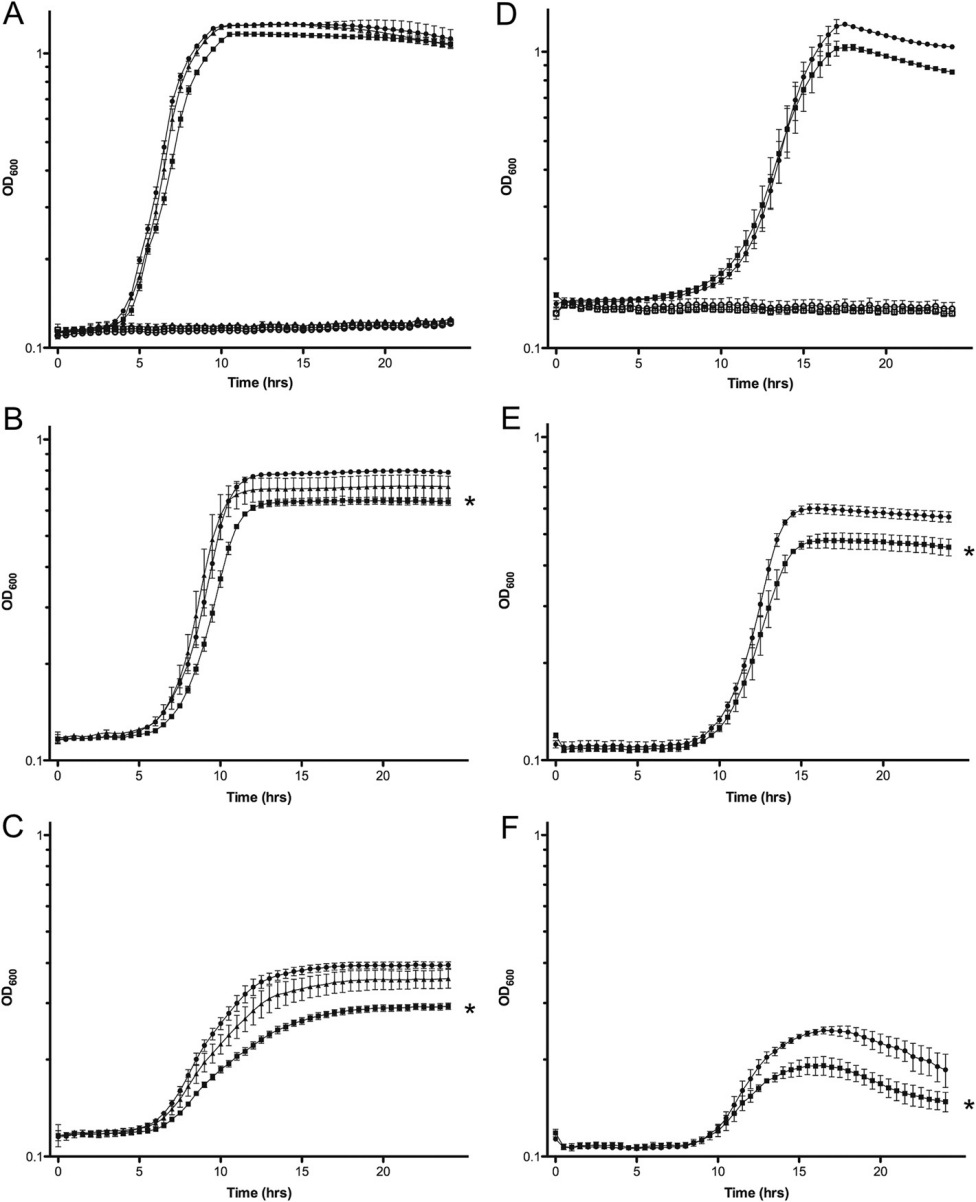
**Growth of nontypable *H. influenzae *strains R2866 and 86**-**028NP *in vitro*.** (**A**-**C**) Growth of R2866 (circles), its isogenic ∆*hfq* mutant derivative (squares) and the complemented ∆*hfq* mutant (triangles). (**D**-**F**) Growth of 86-028NP (circles) and its isogenic ∆*hfq* mutant derivative (squares). (**A** &**D**) Growth in 5 μg mL^-1^ heme (solid symbols and in hdBHI (open symbols). (**B**) Growth of R2866 and its derivatives in 10 μg mL^-1^ hemoglobin. (**C**) Growth of R2866 and its derivatives in 5 μg mL^-1^ hemoglobin. (**E**) Growth of 86-028NP and its derivative in 30 μg mL^-1^ hemoglobin. (**F**) Growth of 86-028NP and its derivative in 20 μg mL^-1^ hemoglobin. The Mann–Whitney test was used to compare make comparisons between strains over the entire 24-hour growth period. For comparisons of the wild type strains with the corresponding mutant in all concentrations of hemoglobin *P<0.0001.

In other bacterial species, Hfq plays a role in iron regulation and tolerance to various stressors, such as oxidative damage, high salt, and detergents [12,20,54,55]. Since *H*. *influenzae* requires heme for aerobic growth, we conducted growth studies to investigate whether the deletion of *hfq* impacted growth and heme source utilization. Direct comparisons were made between each wild type strain, and its ∆*hfq* mutant. The complement strain was also included when studying R2866 and its mutant. Several attempts were made to create a complement for the 86-028NP ∆*hfq* strain, HI2207, but were unsuccessful. Tested heme sources included free heme, hemoglobin, hemoglobin-haptoglobin and heme-hemopexin at various concentrations. The *hfq* mutants of both strains grew at a similar rate to the wild type strains in all growth conditions except under limiting concentrations of hemoglobin (Figure 2). Complementation of the ∆*hfq* mutation did not completely restore the wild type phenotype in R2866, but the complemented strain did grow significantly better than the ∆*hfq* strain. *In vitro* competition experiments were performed in nutrient rich and hemoglobin limiting conditions to determine if competition between the two strains would further inhibit the growth of the ∆*hfq* strain. No difference was observed between the two strains under either growth condition (data not shown). These results suggest that Hfq may be required for *H*. *influenzae* to efficiently utilize certain nutrients from its environment in order to occupy specific niches within the host, as seen in other organisms [18,56]. Previous studies have shown there are two proteins that are required for the uptake of heme from hemoglobin, the TonB-dependent Hgps and Hup proteins [27,57]. However, the expression of these genes is unaffected by the deletion of *hfq* (data not shown). Further studies are needed to understand the potential role of Hfq in the utilization of heme from hemoglobin.

The ability of the *hfq* mutant to tolerate other stressful conditions was also examined. There were no differences observed in growth between the wild type and mutant strains in the presence of oxidative stress induced by the addition of hydrogen peroxide or cumene hydroperoxide (data not shown). Thus, no role was detected for *H*. *influenzae* Hfq in the regulation of genes involved in ameliorating oxidative stress as it does in other bacterial species [12,13]. No significant differences in growth between wild type and mutant strains were seen in media containing high salt or sodium dodecyl sulfate (SDS) at various concentrations (data not shown). In other bacteria Hfq also plays a role in high salt and detergent stress [21]. These data demonstrate that the phenotypic effects in *H*. *influenzae* strains R2866 and 86-028NP lacking *hfq* differ from those observed in other bacterial species [21].

### Role of *hfq* in *H*. *influenzae* pathogenesis

The *hfq* mutants of the nontypeable strains R2866 and 86-028NP were compared for their abilities to establish and maintain infection in two well established animal models of human *H*. *influenzae* disease. The methods used for these studies were designed to test for virulence and fitness of the mutant strains in comparison to their wild type progenitor. The use of different strains is necessary because nontypeable clinical isolates of *H*. *influenzae* generally cannot be used across the different animal models of disease. In our hands, 86-028NP is unable to cause bacteremia in the infant rat model and R2866 infected chinchillas rapidly proceed to inner ear infection and bacteremia, criteria for termination of the experiment (unreported observations). Therefore, in order to compare mutations in multiple animal models, it is necessary to use different *H*. *influenzae* strains.

**Figure 3:**
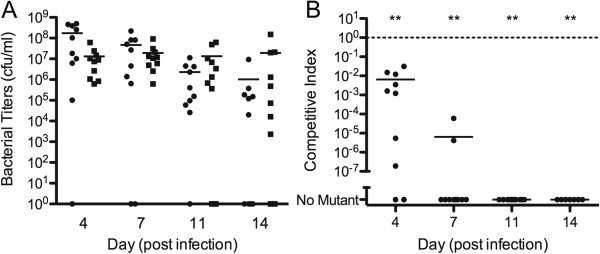
**Deletion of *hfq *in *H. influenzae *strain 86**-**028NP in the chinchilla model of otitis media.** (**A**) Bacterial titers of 86-028NP (closed circles) and the ∆*hfq* strain HI2207 (closed squares) in the middle ear effusions collected on days 4, 7, 11 and 14 post infection. (**B**) Competitive index comparing the input ratios of 86-028NP and HI2207 on day 0 to the output ratios of bacterial titers on the days indicated post infection (**P<0.001).

The nontypeable *H*. *influenzae* strain 86-028NP was compared with the *hfq* mutant HI2207 in the chinchilla model of otitis media. Two separate experiments were performed; a paired comparison assay to determine virulence, and a competition assay to determine fitness defects in the ∆*hfq* strain. In the virulence assay, two groups of five animals were challenged with the wild type and mutant strains, respectively, and assessed on days 4, 7, 11, and 14 post-infection. No differences were observed using video otoscopy and tympanometry between the two groups in the progression of otitis media and there were no qualitative differences in the middle ear effusions (data not shown). Furthermore, there was no statistical difference in bacterial loads in the ear effusions recovered from the two groups (Figure 3A).

In the fitness assays, five chinchillas were challenged with the wild type and mutant strains and disease progression was assessed on days 4, 7, 11, and 14 post-infection (Figure 3B). Over the duration of the experiment, the wild type strain produced titers normally seen in otitis media in the chinchilla following challenge with this strain [46]. However, the mutant strain was unable to compete with wild type in this environment. The average competitive index [(mutant output/WT output)/(mutant input/WT input)] in the ten ears was approximately 0.01 by day four (P<0.001, one sample t-test for competitive index = 1.0) and continued to decline until day 11 when all ears were cleared of the mutant strain (Figure 3B). Because *in vitro* growth rates of mutant and wild type strains were not different in sBHI, the results of the mixed challenge suggest that the mutant’s fitness reduction is specific to the host environment.

**Figure 4:**
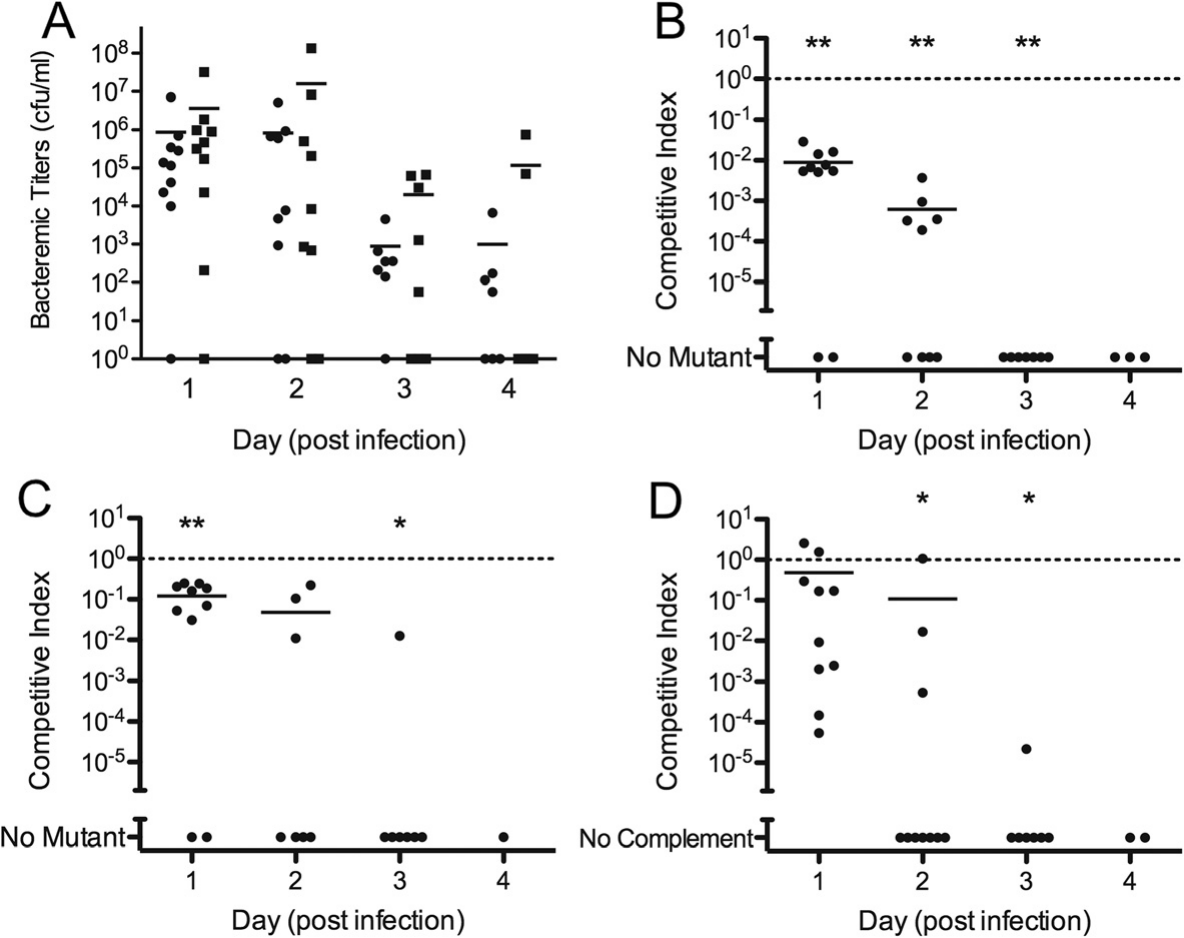
**Comparison of *H. influenzae *strains R2866, HI2206, and HI2210 to sustain bacteremia in infant rats.** (**A**) Bacteremic titers of rats infected with either R2866 (closed circles) or HI2206 (closed squares) in the virulence model of infection. (**B**) Competitive index showing the comparison of bacteria input ratios of R2866 and HI2206 on Day 0 compared to the output ratios on subsequent days of the infection. (**C**) Competitive index comparing the ∆*hfq* strain HI2206 and the complement HI2210. (**D**) Comparison of fitness of R2866 and HI2210. Data are representative of two independent experiments. (**P<0.0001; *P<0.01).

The nontypeable strain R2866 was compared to the *hfq* mutant, HI2206, and the ∆*hfq* complement strain, HI2210, for the ability to establish and maintain bacteremia in the infant rat model of invasive disease. Virulence and fitness models of infection were also used in the infant rats. In the virulence study, two groups of 10 infant rats were infected with the wild type or mutant strain and disease progression was monitored by clinical signs of infection and by bacterial titers in the blood. There was no observed difference in disease progression between the two groups and there was no significant difference in the bacterial titers (Figure 4A).

In the infant rat fitness study, two cohorts of 10 pups were used to compare the fitness of R2866, HI2206, and HI2210. One group of animals was infected with equal numbers of R2866 and HI2206 (Figure 4B). The second group received the complemented strain, HI2210, along with HI2206 (Figure 4C). The last group of animals was infected with R2866 and HI2210 (Figure 4D). The *hfq* mutant exhibited significantly lower bacteremic titers throughout the course of the experiment when compared to either the wild type or the complemented mutant strains. As shown by the competitive index, the ∆*hfq* strain was approximately a 100-fold lower than the wild type strain by day one and all animals had completely cleared the mutant strain by day 3 post infection. Similar differences were observed in the animals infected with the ∆*hfq* complement strain and the ∆*hfq* strain, indicating the complement strain exhibits a reversal of the mutant phenotype, however, there was not a complete reversal of the mutant phenotype (Figure 4D). The wild type strain did significantly out compete the complemented strain on days 2 and 3 post-infection. Complementation only partially restores the *in vitro* growth phenotype, and since the *in vivo* environment is likely to be more rigorously restricted for essential nutrients, the difference between wild type and complemented strain may be exacerbated *in vivo*.

The role of Hfq during infections of *H*. *influenzae* is not clear. In other organisms several sRNAs that interact with Hfq have been shown to be important in the regulation of genes involved in pathogenesis [58]. It is currently unknown if *H*. *influenzae* has sRNAs that are important in pathogenesis. However, our animal studies suggest that in the absence of Hfq, certain genes important in establishing infection are likely affected. Presumably, these genes are regulated by sRNAs either directly or indirectly and require Hfq to function properly. However, during the virulence studies there was no observed difference in either animal model, indicating that the ∆*hfq* mutant was able to grow within the host environment. The defect is apparently limited to the occupation of specific niches within the host that are unavailable in a mixed infection due to the presence of the wild type strain. The loss of post-transcriptional regulation in the ∆*hfq* mutant leads to the inability of the bacteria to adapt to the host environment and compete successfully for the specific niches that are required for pathogenesis. The observations made in this study indicate there is a decrease in fitness in the animal models, and this phenotype is conserved across different strains. This effect may be partially explained by the impact of *hfq* mutation on acquisition of essential nutrients such as heme. While we did not address biofilm formation in the chinchilla middle ear, the possibility remains that mutation of *hfq* may influence adherence/biofilm formation in the microenvironment. A better understanding of the nutrients available in the host is necessary for a comprehensive explanation of the decrease in fitness identified in the mutant strain.

## Conclusion

The growth and survival of pathogenic bacteria critically depends on their ability to monitor environmental changes within the host organism and adjust their own expression of stress- and virulence-associated genes accordingly. The RNA chaperone Hfq is important in modulating genes essential to stress and virulence in a variety of bacterial pathogens by binding sRNAs and their mRNA target [14,51,59]. Our study is the first to report the role of Hfq in *H*. *influenzae* and highlights the impact of Hfq on nutrient acquisition *in vitro* and infection progression *in vivo* of this important human pathogen.

## Competing interests

The authors declare that they have no competing interests.

## Authors’ contributions

RJH conceived the study. All authors participated in the study design and data analysis. RJH performed the sequence alignment, primer extension assay, *in vitro* growth assays, and drafted the manuscript. RJH and DJM performed the chinchilla experiments. RJH and TWS performed the infant rat studies. DJM, TWS, PWW and TLS revised the manuscript. All authors read and approved the final manuscript.
